# Fecal microbiota transplantation and short‐chain fatty acids protected against cognitive dysfunction in a rat model of chronic cerebral hypoperfusion

**DOI:** 10.1111/cns.14089

**Published:** 2023-01-10

**Authors:** Shao‐Hua Su, Ming Chen, Yi‐Fang Wu, Qi Lin, Da‐Peng Wang, Jun Sun, Jian Hai

**Affiliations:** ^1^ Department of Neurosurgery, Tongji Hospital, School of Medicine Tongji University Shanghai China; ^2^ Department of Neurosurgery, Xinhua hospital, School of Medicine Shanghai Jiao Tong University Shanghai China; ^3^ Department of Pharmacy, Institutes of Medical Sciences, School of Medicine Shanghai Jiao Tong University Shanghai China

**Keywords:** chronic cerebral hypoperfusion, cognitive dysfunction, fecal microbiota transplantation, microglia, oxidative phosphorylation, short‐chain fatty acids, synaptic plasticity

## Abstract

**Aims:**

Clear roles and mechanisms in explaining gut microbial dysbiosis and microbial metabolites short‐chain fatty acids (SCFAs) alterations in chronic cerebral ischemic pathogenesis have yet to be explored. In this study, we investigated chronic cerebral hypoperfusion (CCH)‐induced gut microbiota and metabolic profiles of SCFAs as well as the effects and mechanisms of fecal microbiota transplantation (FMT) and SCFAs treatment on CCH‐induced hippocampal neuronal injury.

**Methods:**

Bilateral common carotid artery occlusion (BCCAo) was used to establish the CCH model. Gut microbiota and SCFAs profiles in feces and hippocampus were evaluated by 16S ribosomal RNA sequencing and gas chromatography–mass spectrometry. RNA sequencing analysis was performed in hippocampal tissues. The potential molecular pathways and differential genes were verified through western blot, immunoprecipitation, immunofluorescence, and ELISA. Cognitive function was assessed via the Morris water maze test. Ultrastructures of mitochondria and synapses were tested through a transmission electron microscope.

**Results:**

Chronic cerebral hypoperfusion induced decreased fecal acetic and propionic acid and reduced hippocampal acetic acid, which were reversed after FMT and SCFAs administration by changing fecal microbial community structure and compositions. Furthermore, in the hippocampus, FMT and SCFAs replenishment exerted anti‐neuroinflammatory effects through inhibiting microglial and astrocytic activation as well as switching microglial phenotype from M1 toward M2. Moreover, FMT and SCFAs treatment alleviated neuronal loss and microglia‐mediated synaptic loss and maintained the normal process of synaptic vesicle fusion and release, resulting in the improvement of synaptic plasticity. In addition, FMT and SCFAs supplement prevented oxidative phosphorylation dysfunction via mitochondrial metabolic reprogramming. The above effects of FMT and SCFAs treatment led to the inhibition of CCH‐induced cognitive impairment.

**Conclusion:**

Our findings highlight FMT and SCFAs replenishment would be the feasible gut microbiota‐based strategy to mitigate chronic cerebral ischemia‐induced neuronal injury.

## Graphical abstract

Fecal microbiota transplantation (FMT) and short‐chain fatty acids (SCFAs) replenishment exerted anti‐neuroinflammatory effects through switching microglial phenotype from M1 toward M2. Furthermore, FMT and SCFAs treatment alleviated neuronal loss and microglia‐mediated synaptic loss and maintained the normal process of synaptic vesicle fusion and release, resulting in the improvement of synaptic plasticity. In addition, FMT and SCFAs supplement prevented oxidative phosphorylation dysfunction via mitochondrial metabolic reprogramming.

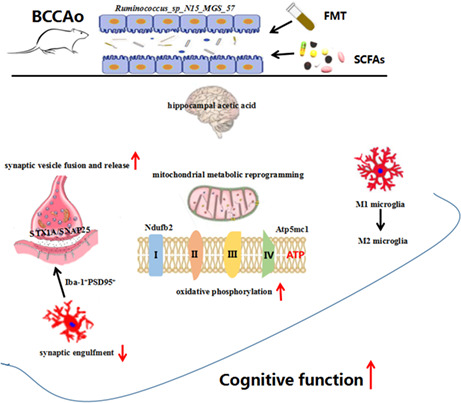

## 1 INTRODUCTION

Recent evidence indicates that the gut microbiota (GM) regulate the development and function of the immune, metabolic, and nervous systems through dynamic bidirectional communication along the microbiota‐gut‐brain axis.^1^ Compositional changes to the GM are known to influence the homeostasis of the host and are closely associated with multiple diseases related to the central never system (CNS), including Alzheimer's disease, Parkinson's disease, multiple sclerosis, autism, and psychiatric disorders.^2^, ^3^, ^4^, ^5^, ^6^ Nevertheless, the roles of GM in CNS‐related diseases remain unclear.

Alterations to the gut microbiome have been linked to microglia‐mediated neuroinflammation and cognitive impairment caused by intracranial ischemic injury^7^, ^8^ and are recognized as potential risk factors for acute cerebral ischemia (CI) and stroke.^9^ Fecal microbiota transplantation (FMT) is reportedly an effective treatment for acute CI,^10^ although relatively little is known about the association between GM and chronic CI. Notably, a previous study confirmed persistent dysbiosis of the GM at 12 months after cerebral infarction in cynomolgus monkeys.^11^ However, the long‐term therapeutic outcomes of FMT for chronic CI have not yet been clarified, thus further investigations are warranted.

According to previous studies,^12^, ^13^ the dominant phyla of the GM of both the rat and humans include *Firmicutes*, *Bacteroidetes*, *Proteobacteria*, and *Actinobacteria*. In addition, *Lactobacillus* and *Turicibacter* are the dominant genera of the rat GM, but not *Bifidobacterium* or *Faecalibacterium*. Species of GM could be either pathogenic or commensal, depending on environmental factors and disease status. Also, the same species could promote the development of one disease, while protecting against another.^14^ However, the effects of GM on chronic CI are largely unknown.

Short‐chain fatty acids (SCFAs) produced by bacteria mainly include acetate, propionate, and butyrate, which can cross the blood–brain barrier (BBB) and significantly influence brain processes owing to various neuroactive properties and effects on the gut‐brain signaling pathways.^15^, ^16^ SCFAs are reported to modulate brain function through the immune, endocrine, vagal, and humoral pathways,^17^ and influence neuroinflammation, secretion of gut hormones and neurotrophic factors, as well as activation of vagal afferents that either directly or indirectly affect neural functioning, learning, memory, and mood. SCFAs have also been found to alleviate neuroinflammation and neurological deficits after ischemic stroke,^18^ thus presenting a potentially effective treatment for CI.^10^ However, SCFAs were shown to significantly impact dysbiosis of the GM, indicating that the effects of SCFAs on CI‐related stroke are controversial. In addition, relatively few studies have explored the effects of SCFAs as potential mediators of GM‐targeted interventions against chronic CI. A previous study demonstrated that SCFAs produced by the GM were significantly decreased at 6 and 12 months after cerebral infarction in cynomolgus monkeys.^11^ Hence, further elucidation of the effects of SCFAs produced by the GM could expand microbial‐based strategies for the treatment of chronic CI.

Histone deacetylases (HDACs) play crucial roles in diseases of the CNS and are classified as class I (HDAC1–3, 8), II (HDAC4–7, 9, 10), III (Sirtuin1–7), and IV (HDAC11).^19^ In the hippocampus, HDAC1, HDAC2, and HDAC8 are reported to enhance neuroinflammation related to cognitive dysfunction,^20^ while HDAC3 reduces dendritic spine density and levels of proteins related to synaptic plasticity.^21^ In addition, increased activity of HDAC6 has been associated with memory impairment.^22^ In contrast, the expression levels and activities of HDAC4 and HDAC5 are down‐regulated in the hippocampus after mild traumatic brain injury.^23^ Furthermore, long‐term treatment with acetate was shown to decrease mRNA levels of HDAC2, HDAC5, HDAC7, and HDAC8,^24^ suggesting that SCFAs mediate the expression of HDACs. In chronic CI, HDACs inhibition may be the most important effect of SCFAs, although further investigations are needed to confirm this hypothesis.

Chronic CI refers to a prolonged decrease in cerebral blood flow, which is considered to be maintained by chronic cerebral hypoperfusion (CCH). In the present study, the GM and metabolic profiles of SCFAs in response to hippocampal injury were investigated using a rat model of CCH, which mimics a state of chronic CI in humans. Furthermore, FMT and supplementation of SCFAs were applied to assess the potential roles and mechanisms of the GM and SCFAs for the treatment of CCH‐induced hippocampal injury to design new and effective therapies for chronic CI.

## 2 MATERIALS AND METHODS

### 2.1 Animals and experimental protocols

**FIGURE 1:**
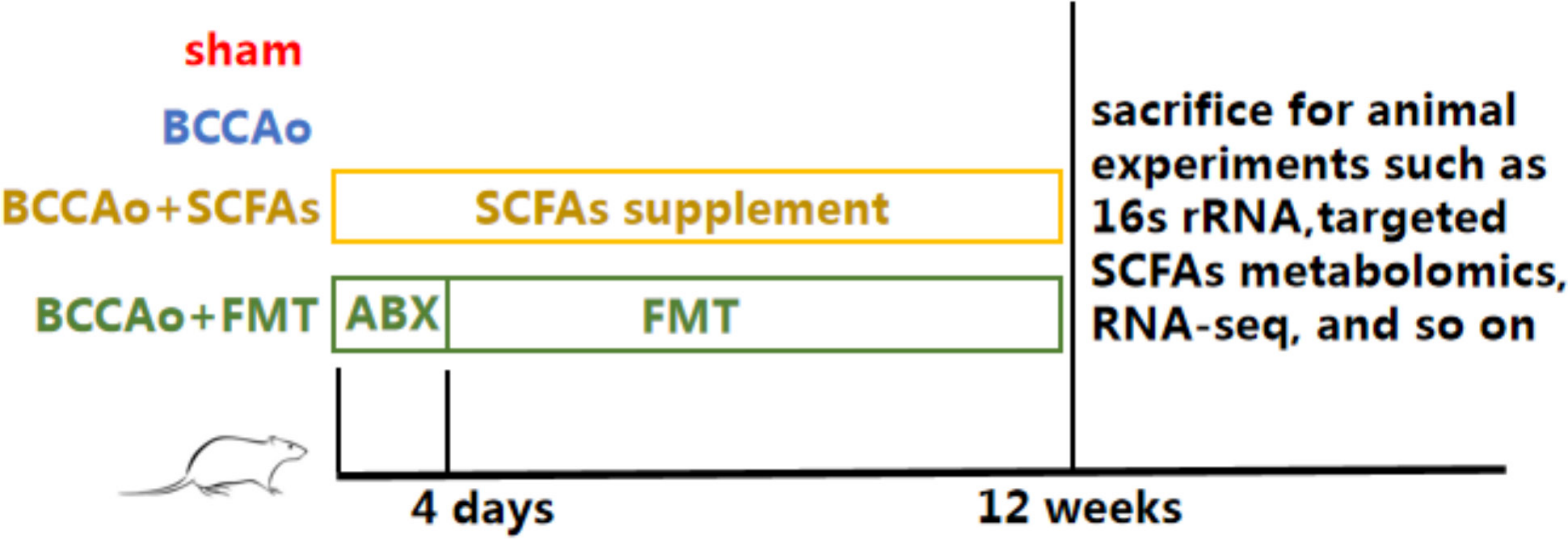
Experimental time schedule. After 1 week of acclimatization to the laboratory conditions, rats were randomly divided into sham, BCCAo, BCCAo + FMT, and BCCAo + SCFAs groups. Rats were sacrificed for experiments after receiving different interventions in four groups for 12 weeks. ABX, antibiotic treatment; BCCAo, bilateral common carotid artery occlusion; FMT, fecal microbiota transplantation; SCFAs, short‐chain fatty acids.

The protocols of all animal studies were approved by the Institutional Animal Care and Use Committee of Tongji Hospital and conducted in accordance with the Guide for the Care and Use of Laboratory Animals. Male Sprague–Dawley rats (age, 5 weeks; body weight [BW], 180 ± 10 g) were purchased from the Shanghai Laboratory Animals Research Center (Shanghai, China) or Sino‐British Sippr/BK Lab Animal Co., Ltd. (Shanghai, China) and housed in a specific pathogen‐free animal facility under a 12‐h light/dark cycle at 23 ± 1°C and constant humidity of 60% with ad libitum access to food and water. After a 1‐week acclimation period, the rats were randomly allocated to one of four groups: (1) sham‐operated group, (2) bilateral common carotid artery occlusion (BCCAo) group, (3) BCCAo + FMT group, or (4) BCCAo + SCFAs group. The experimental time schedule is shown in Figure 1.

At week 12, the rats were sacrificed after assessment of spatial learning with the Morris water maze. Fresh fecal samples and brain tissues were immediately collected for analysis or stored at −80°C.

### 2.2 BCCAo procedure

At 8–12 weeks after BCCAo, the CCH phase was maintained in the rats to mimic decreased cerebral blood flow in humans.^25^ Hence, in the present study, BCCAo was continued for 12 weeks to simulate chronic CI. Afterward, the rats were anesthetized by intraperitoneal injection of pentobarbital sodium at 50 mg/kg BW. The bilateral common carotid arteries were exposed through a midline incision and subsequently tightly double‐ligated with 5‐0 silk sutures. The sham rats underwent the same procedure as the BCCAo rats but without ligation of the bilateral common carotid arteries.

### 2.3 Antibiotic, FMT, and SCFAs treatment

Rats in the BCCAo + FMT group were given an antibiotic cocktail consisting of 100 mg/kg BW of vancomycin, 200 mg/kg BW of neomycin sulfate, 200 mg/kg BW of metronidazole, and 200 mg/kg BW of ampicillin by gastric gavage daily for 4 days to deplete the GM.^10^

Fresh fecal samples were collected from rats in the sham group, then immediately diluted with sterile physiological saline solution to 100 mg/mL and centrifuged at 8000 × *g* and 4°C for 5 min to obtain the final GM suspension. Each rat in the BCCAo + FMT group was intragastrically administrated 2 ml of the final GM suspension for 12 consecutive days.^26^ Furthermore, the above process was performed once every 3 days for 12 weeks to maintain the effects of FMT.

Rats in the BCCAo + SCFAs group were administrated a cocktail of SCFAs consisting of 67.5 mM sodium acetate, 25.9 mM sodium propionate, and 40 mM sodium butyrate (Sigma‐Aldrich Corporation, St. Louis, MO, USA) by gastric gavage at 0.1 ml/10 g BW daily for 12 consecutive weeks.^27^, ^28^

### 2.4 16S ribosomal RNA (rRNA) gene sequencing

Frozen fecal samples were sent to BioNovoGene Co., Ltd. (Suzhou, China) for sequencing of the 16S rRNA gene. Briefly, total DNA was extracted from the fecal samples (250 mg, wet weight) using a QIAamp DNA Stool Mini Kit (Qiagen GmbH, Hilden, Germany) in accordance with the manufacturer's instructions. For each DNA sample, the V3 and V4 hypervariable regions of the 16S rRNA gene were amplified with the primer pair 341F (5′‐CCT AYG GGR BGC ASC AG‐3′)/806R (5′‐GGA CTA CNN GGG TAT CTA AT‐3′). Then, the PCR products were purified using the AxyPrep™ DNA Gel Extraction Kit (Axygen Scientific, Inc., Union City, CA, USA). Afterward, the purified PCR products were pooled in equal molar concentrations, quantified with a QuantiFluor™‐ST fluorometer (Promega Corporation, Madison, WI, USA), and sequenced using a NovaSeq PE250 sequencing instrument (Illumina, Inc., San Diego, CA, USA) in accordance with the manufacturer's instructions.

Quantitative Insights Into Microbial Ecology software (version 1.9.1) was used for the analysis of the 16S rRNA sequences.^29^ The Chao1 richness and abundance‐based coverage estimator (ACE) diversity indices were calculated to evaluate the diversity of GM species within samples and compared with the Mann–Whitney U test. Principal coordinates analysis was used to assess the diversity of GM species between samples. Microbiota dysbiosis was assessed by calculating the relative abundance of each taxonomic group of the GM at the family and species levels. Linear discriminant analysis with effect size was used to identify differences in the abundance of GM species. These data can be accessed at the BioProject database (identification code: PRJNA869931).

### 2.5 SCFAs profiling

The amounts of SCFAs in fecal samples (100 mg) and hippocampal tissues (60 mg) were determined by gas chromatography–mass spectrometry (GC–MS) with a TRACE™ 1310‐ISQ LT GC system (Thermo Fisher Scientific, Waltham, MA, USA) and conducted by BioNovoGene Co., Ltd. The standards of SCFAs were mixtures of acetate, propionate, butyrate, isobutyrate, valerate, isovalerate, and caproate. Helium was used as a carrier gas at a constant flow rate of 1 ml/min. Briefly, samples (1 μl) were injected at a split ratio of 10:1. The temperatures of the injector, transfer line, and ion source were set to 250°C, 250°C, and 230°C, respectively. The initial temperature was maintained at 90°C for 2 min and then increased to 120°C at 10°C/min, 150°C at 5°C/min, and 250°C at 25°C/min. The final temperature was maintained for 2 min. Data acquisition was performed with an electron impact ionization of 70 eV in a full‐scan mode with an m/z range of 35–780.

Normalization, transformation, outlier removal, and scaling of the GC–MS data were conducted with R software (v3.3.2). The concentrations of seven metabolites of the SCFAs were quantified and compared with the nonparametric Mann–Whitney U test. Partial least squares discriminate analysis was used to identify differences among the groups.

### 2.6 RNA sequencing

RNA‐seq of frozen hippocampal tissues was conducted by Suzhou BioNovoGene Co., Ltd. Briefly, RNA was purified using the RNeasy Mini kit (Qiagen GmbH). The purified RNA was sequenced with the NovaSeq Pe250 Platform (Illumina Inc.) in accordance with the manufacturer's instructions. Gene set enrichment analysis was performed in reference to the Kyoto Encyclopedia of Genes and Genomes and the Gene Ontology database. A differentially expressed gene (DEG) was based on the following criteria: probability (*p*) value <0.05 and |log_2_ (fold change)| > 0.5. These data can be accessed at the BioProject database (identification code: PRJNA827266).

### 2.7 Morris water maze

Briefly, a circular pool (diameter, 1.8 m; height, 60 cm) was divided into four quadrants designated as north, south, east, and west. Rats were trained four times per day for four consecutive days. During the training period, a white platform (diameter, 9 cm) was placed 1 cm below the surface of the water in the middle of the southwest quadrant. During each trial, rats were allowed a maximum of 60 s to arrive at the platform. Rats that successfully found the platform were kept on the platform for 15 s, while the other rats were manually guided to the platform. The latency to escape was calculated as the average time to find the platform during the four trials conducted on the same day. On day 5, the test was conducted without the platform, which forced each rat to swim freely in the pool for 60 s. The percentage of time in the platform quadrant, the number of platform crossings, swimming speed, and swimming paths were measured. All data were recorded with a computerized video system.

### 2.8 Mitochondrial isolation

Mitochondria were isolated with a Qproteome Mitochondria Isolation Kit (Qiagen GmbH). Briefly, hippocampal tissues (20 mg) were cut into pieces, homogenized in 2 ml of lysis buffer with protease inhibitor solution, and centrifuged at 1000 *g* for 10 min at 4°C. Then, the supernatant was collected, resuspended in 1.5 ml of ice‐cold disruption buffer, and centrifuged at 6000 *g*. The second supernatant was collected, resuspended in 750 μl of mitochondrial purification buffer, and added on top of a mitochondrial purification buffer layer. After centrifugation at 14,000 *g* for 15 min, the pellet containing the mitochondria was washed three times with 1.5 ml of mitochondrial storage buffer via centrifugation at 8000 *g* for 10 min. Finally, the purified mitochondria were resuspended in a mitochondrial storage buffer for detection of membrane potential or stored at −80°C for later analysis.

### 2.9 Mitochondrial membrane potential

Mitochondria membrane potential was measured using a JC‐1 staining kit (Beyotime Institute of Biotechnology, Shanghai, China). Briefly, high‐purity mitochondria (10 μl) were incubated in JC‐1 staining reagent (100 μl) for 10 min. Images were captured with a fluorescence microscope (IX71; Olympus Corporation, Tokyo, Japan). The ratio of red to green fluorescence was calculated to evaluate the membrane potential of isolated mitochondria among the different groups. Healthy mitochondria, mostly in the first quadrant of the flow analysis diagram, showed a high intensity of red fluorescence and a low intensity of green fluorescence, while injured mitochondria, mostly in the fourth quadrant of the flow analysis diagram, displayed low intensity of red fluorescence, and high intensity of green fluorescence.

### 2.10 Adenosine triphosphate (ATP) content and electron transport chain (ETC) complex I–V activities of the hippocampal tissues

The ATP content and ETC complex I–V activities of the hippocampal tissues were quantified using commercial assay kits (Beijing Solarbio Science & Technology Co., Ltd., Beijing, China) in accordance with the manufacturer's instructions. Briefly, equal amounts of hippocampal proteins were loaded into the wells and the absorbance was measured with a spectrophotometer at 340 nm for nicotinamide adenine dinucleotide NADH dehydrogenase (complex I) and ATP content, 550 nm for cytochrome c reductase (complex III) or cytochrome c oxidase (complex IV), 605 nm for succinate‐coenzyme Q reductase (complex II), and 660 nm for F_0_F_1_ ATPase (complex V). ATP content is expressed as μmol/mL, while ETC complex I–V activities are expressed as μmol/mg protein.

### 2.11 Dihydroethidium (DHE) staining

Reactive oxygen species (ROS) were detected by staining with DHE. Briefly, the tissue samples were incubated in 10 mM DHE (Beyotime Institute of Biotechnology) at room temperature for 30 min in the dark and then observed under an inverted microscope (IX71; Olympus Corporation). The DHE staining results were confirmed by flow cytometry.

### 2.12 Immunofluorescence labeling

Briefly, brain tissues were fixed in 4% paraformaldehyde at 4°C overnight and subsequently divided along the midline. The hemisphere sections were serially dehydrated, embedded in paraffin, and then cut into 5 μm‐thick coronal sections for immunofluorescence. After antigen retrieval with Tris‐ethylenediaminetetraacetic acid buffer solution (pH 9.0), the deparaffinized sections were incubated with 10% non‐immune goat serum for 30 min at room temperature, followed by primary antibodies against the neuronal nuclear antigen (dilution, 1:200; Abcam, Cambridge, MA, USA), ionized calcium‐binding adapter molecule 1 (Iba‐1) (dilution, 1:250; Abcam), interleukin‐1β (IL‐1β) (dilution, 1:250; Invitrogen Corporation, Carlsbad, CA, USA), arginase 1 (Arg‐1) (dilution, 1:250; Proteintech, Rosemont, IL, USA), postsynaptic density 95 (PSD95) (dilution, 1:200; Cell Signaling Technology, Inc., Danvers, MA, USA), and glial fibrillary acidic protein (GFAP) (dilution, 1:200; Abcam) at 4°C overnight in a humidified chamber. After washing with phosphate‐buffered saline, the sections were stained with tetraethylrhodamine isothiocyanate‐conjugated secondary antibodies (dilution, 1:200; Santa Cruz Biotechnology, Inc., Dallas, TX, USA) for 1 h at 37°C. Positive cells in the hippocampal CA1 region were visualized with a confocal fluorescence microscope (LSM 700; Carl Zeiss AG, Jena, Germany).

### 2.13 Western blot analysis

The hippocampal tissues were homogenized in an ice‐cold buffer and then centrifuged at 10,000 *g* for 5 min at 4°C. Then, the supernatants were collected for western blot analysis. Briefly, protein samples (20 μg) were separated by electrophoresis with 8%, 10%, and 12% polyacrylamide gels, then electroblotted onto polyvinylidene difluoride membranes, which were probed with antibodies against HDAC6 (dilution, 1:1500; Abcam), occludin (dilution, 1:2000; Abcam), claudin 5 (dilution, 1:500; Invitrogen Corporation), early growth response 1 (Egr1) (dilution, 1:1000; Cell Signaling Technology, Inc.), brain‐derived neurotrophic factor (BDNF) (dilution, 1:1500; Abcam), vesicle‐associated membrane protein 2 (VAMP2) (dilution, 1:1000; Abcam), synaptosomal‐associated protein 25 (SNAP25) (dilution, 1:1000; Abcam), syntaxin 1a (Stx1a) (dilution, 1:1500; Abcam), growth associated protein 43 (Gap43) (dilution, 1:1500; Abcam), synaptophysin (Syp) (dilution, 1:1000; Abcam), N‐methyl‐D‐aspartate receptor 1 (NMDAR1) (dilution, 1:1000; Abcam), PSD95 (dilution, 1:1000; Cell Signaling Technology, Inc.), NADH:ubiquinone oxidoreductase subunit B2 (Ndufb2) (dilution, 1:500; Abcam), ATP synthase membrane subunit c locus 1 (Atp5mc1) (dilution, 1:500; Abcam), voltage dependent anion channel 1 (VDAC1) (dilution, 1:1000, Santa Cruz Biotechnology, Inc.), and β‐actin (dilution, 1:5000; Abcam) overnight at 4°C followed by horseradish peroxidase‐conjugated goat anti‐rabbit or mouse immunoglobulin G secondary antibody for 1 h at room temperature. Detection of the protein bands was performed with an enhanced chemiluminescent substrate solution (EMD Millipore Corporation, Billerica, MA, USA). The proteins were quantified against the optical density of β‐actin or VDAC1 as an internal control.

### 2.14 Immunoprecipitation (IP) analysis

Briefly, homogenated hippocampal tissues (200 μg) were incubated with Stx1a antibody (3 μg) at 4 °C overnight. After washing three times with IP buffer for 15 min, the proteins were incubated with protein G‐sepharose beads (Sigma‐Aldrich Corporation) and the antibody mixture for 2 h at 4°C. Subsequently, the precipitants were centrifuged at 10,000 *g* for 1 min and washed three times with IP buffer to remove nonspecifically bound proteins. Afterward, the immune‐complexed beads were resuspended in a loading buffer, heated at 95°C for 5 min, and centrifuged at 10,000 *g*. Finally, the supernatants were applied for immunoblot detection of Stx1a and SNAP25. The remaining homogenates were used as input controls.

### 2.15 Enzyme‐linked immunosorbent assay (ELISA)

The amount of acetyl‐coenzyme A (acetyl‐CoA) in hippocampal tissues was quantified with a commercial ELISA kit (Nanjing Jiancheng Bioengineering Institute, Nanjing, China) in accordance with the manufacturer's instructions. Briefly, equal amounts of proteins were loaded into the wells and the optical density was measured at a wavelength of 450 nm and compared to a standard curve. The results are expressed as ng/ml protein.

### 2.16 Electron microscopy

For observation of ultrastructural changes to the synapses and mitochondria, fresh 1 mm‐thick coronal slices in the hippocampal CA1 area were fixed in 2.5% glutaraldehyde overnight at 4°C, washed three times with 0.1 M phosphate‐buffered saline, fixed in 1% osmium tetroxide for 2 h at 4°C, then dehydrated with a graded series of ethanol and embedded in epoxy resin. Randomly selected ultrathin sections were stained with uranyl acetate and lead citrate and observed under an electron microscope (Philips Healthcare, Best, Netherlands).

### 2.17 Statistical analysis

The data are presented as the mean ± standard error of the mean. Comparisons were conducted using a one‐way analysis of variance, followed by the post hoc Dunnett's test for multiple comparisons. A *p*‐value <0.05 was considered statistically significant.

## 3 RESULTS

### 3.1 FMT and SCFAs ameliorated CCH‐induced dysbiosis of the GM

**FIGURE 2:**
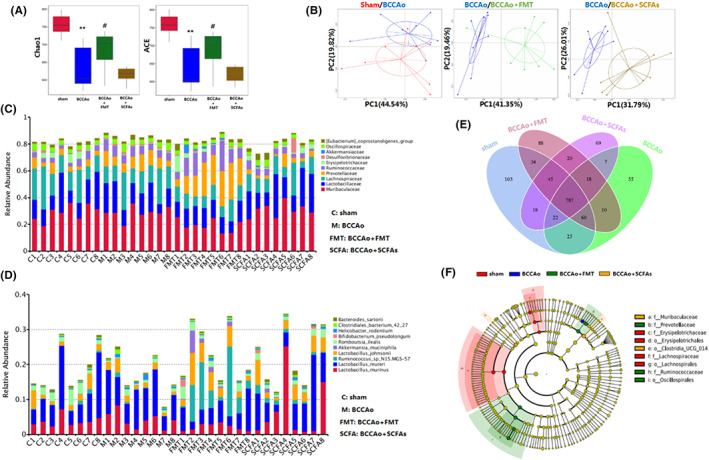
Fecal microbiota transplantation and short‐chain fatty acids treatment reversed CCH‐induced gut microbial dysbiosis. (A) Analysis of alpha diversity via Chao 1 richness and ACE index. (B) Analysis of beta diversity via principal coordinates analysis (PCoA). (C) Relative abundance of top 10 gut microbiota from each sample at the family level. (D) Relative abundance of top 10 gut microbiota from each sample at the species level. (E) Venn diagram showing the shared or unique OTUs by groups. (F) Cladogram generated from linear discriminant analysis (LDA) with effect size (LEfSe). Data are expressed as mean ± SEM (*n* = 8 per group). *p < 0.05 versus sham group. #*p* < 0.05 versus BCCAo group.

The results were shown in our previous study,^30^ at 12 weeks after BCCAo, the Chao1 richness and ACE diversity indices had significantly decreased (both, *p* < 0.001) in the BCCAo group as compared to the sham group, indicating that the diversity of the GM had gradually decreased with prolonged CI (Figure 2A). However, FMT alone had significantly increased the Chao1 richness and ACE diversity indices (*p* = 0.019 and 0.034, respectively), suggesting the positive effects of FMT against the CCH‐induced reduction of the GM. Beta diversity, as determined by principal coordinates analysis based on the Bray–Curtis unweighted unique fraction metric distance reflects differences in the GM composition, which showed clear separate grouping patterns between the sham and BCCAo groups (ADONIS, *p* = 0.002), BCCAo and BCCAo + FMT groups (ADONIS, *p* < 0.001), and BCCAo and BCCAo + SCFAs groups (ADONIS, *p* < 0.001) (Figure 2B). Furthermore, Venn diagrams created to show the number of shared or unique operational taxonomic units (OTUs) among the four treatment groups identified 103/1094 unique OTUs in the sham group, 55/984 in the BCCAo group, 88/1062 in the BCCAo + FMT group, and 69/986 in the BCCAo + SCFAs group at 12 weeks after BCCAo (Figure 2E). Moreover, differences in taxa abundance among the four groups were assessed at the family and species levels. Detailed analysis of the top 10 taxa at the family level demonstrated that FMT or SCFAs significantly increased the relative abundance of *Prevotellaceae* (BCCAo vs. BCCAo + FMT, *p* = 0.003), *Ruminococcaceae* (BCCAo vs. BCCAo + FMT, *p* = 0.017), *Erysipelotrichaceae* (BCCAo vs. BCCAo + SCFA, *p* = 0.004), and *Akkermansiaceae* (BCCAo vs. BCCAo + FMT, *p* = 0.001; BCCAo vs. BCCAo + SCFAs, *p* = 0.001), while specific analysis of top 10 intestinal bacteria at the species level demonstrated that FMT or SCFAs markedly increased the relative abundance of *Lactobacillus johnsonii* (BCCAo vs. BCCAo + FMT, *p* = 0.03), *Ruminococcus_sp_N15_MGS_57* (BCCAo vs. BCCAo + FMT, *p* = 0.002), and *Akkermansia muciniphila* (BCCAo vs. BCCAo + FMT, *p* = 0.001; BCCAo vs. BCCAo + SCFAs, *p* = 0.001) at 12 weeks after BCCAo (Figure 2C,D). Additionally, linear discriminant analysis was performed to further investigate alterations to the GM biomarkers among the four groups, which showed that FMT and SCFAs enriched the populations of *Ruminococcaceae* at the family level and *Clostridia_UCG_014* at the order level (Figure 2F).

Taken together, these results suggest that CCH triggered changes to the composition of the GM, while FMT and SCFAs remodeled the GM after chronic CI by enriching the abundance of *Ruminococcaceae* And *Clostridia_UCG_014*.

### 3.2 FMT and SCFAs alleviated the CCH‐induced decrease in acetic acid content by regulating the GM

**FIGURE 3:**
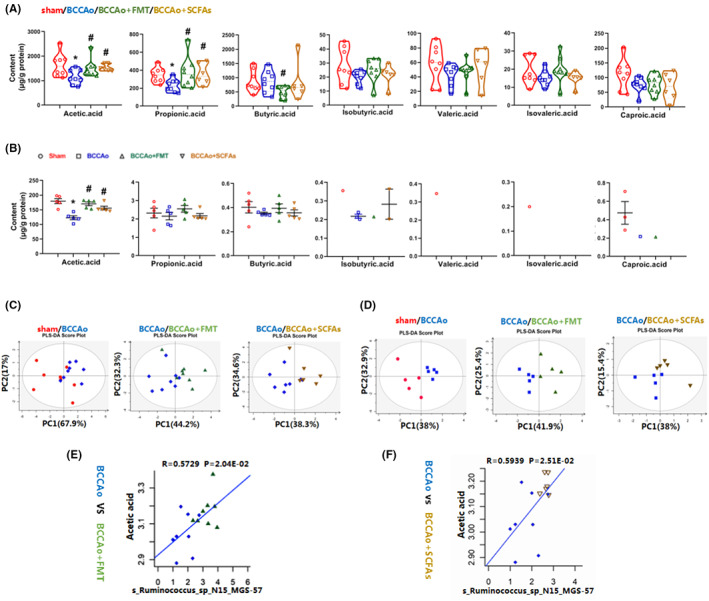
FMT and SCFAs treatment altered CCH‐induced SCFAs profiles. (A) Fecal SCFAs concentration determined by gas chromatography–mass spectrometry (GC–MS; *n* = 8 in sham, BCCAo, BCCAo + FMT group; *n* = 6 in BCCAo + SCFAs group). (B) Hippocampal SCFAs concentration determined by GC–MS (*n* = 5 per group). Isobutyric acid, valeric acid, isovaleric acid, and caproic acid were not detected in all the hippocampal samples. (C, D) Segregation trends of fecal and hippocampal SCFAs were assessed via partial least squares discriminant analysis (PLS‐DA), respectively. (E, F) Spearman correlation analysis between *Ruminococcus_sp_N15_MGS_57* and acetic acid from fecal samples. Data are expressed as mean ± SEM. *p < 0.05 versus sham group. #*p* < 0.05 versus BCCAo group. BCCAo, bilateral common carotid artery occlusion; CCH, chronic cerebral hypoperfusion; FMT, fecal microbiota transplantation; SCFAs, short‐chain fatty acids.

Microbial metabolites of SCFAs have been proposed as important regulators of the microbiota‐gut‐brain axis. Hence, the concentrations of SCFAs in the fecal samples and hippocampal tissues among the different groups were detected by GC–MS. Partial least squares discriminate analysis clearly demonstrated differences in the microbial metabolites of SCFAs among the groups (Figure 3C,D). The acetic and propionic acid contents were decreased in the fecal samples after BCCAo (*p* = 0.036 and 0.009, respectively), which were reversed following FMT and treatment with SCFAs (*p* = 0.046/0.028 and 0.036/0.02, respectively) (Figure 3A).^30^ Intriguingly, only acetic acid was obviously diminished in the hippocampal tissues after BCCAo (*p* = 0.008), which was restored to normal levels after FMT and treatment with SCFAs (*p* = 0.008 and 0.008, respectively) (Figure 3B), suggesting that the greatest change among the metabolites in the hippocampal tissues occurred with acetic acid after FMT and treatment with SCFAs for CCH.

A previous study reported that the production of metabolites of SCFAs was strongly associated with the GM composition.^31^ To further explore whether increased production of acetic acid was due to alterations to the GM after FMT and treatment with SCFAs in a state of chronic CI, combined analysis of fecal 16S rRNA, and GC–MS of the metabolites of SCFAs was subsequently conducted. The results showed that an increase in acetic acid content was positively associated with the increased abundance of *Ruminococcus_sp_N15_MGS_57* after FMT and treatment with SCFAs (*p* = 0.02 and 0.025, respectively) (Figure 3E,F),^30^ indicating that the increased production of acetic acid likely resulted from the increased abundance of *Ruminococcus_sp_N15_MGS_57* in response to CCH.

### 3.3 FMT and SCFAs facilitated hippocampal recovery through regulation of histone modifications, synaptic function, and mitochondrial energy metabolism

**FIGURE 4:**
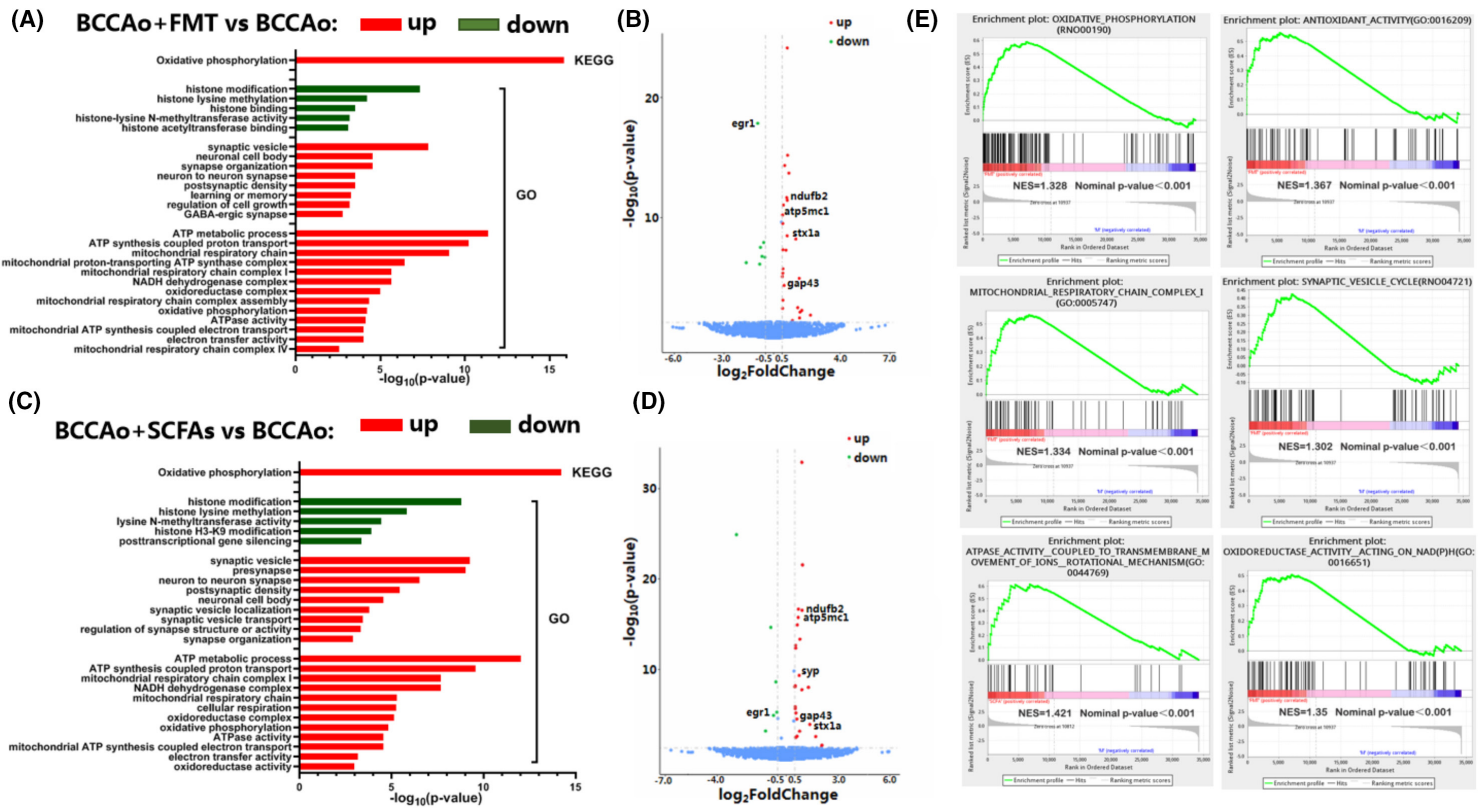
The potential molecular mechanisms involved in the FMT and SCFAs treatment in the hippocampus based on RNA sequencing. (A, C) Histogram of the significantly enriched GO or KEGG terms in the BCCAo group versus BCCAo + FMT group and BCCAo group versus BCCAo + SCFAs group, respectively. (B, D) Volcano plots showing partial differentially expressed genes (DEGs) involved in the above pathways in the BCCAo group versus BCCAo + FMT group and BCCAo group versus BCCAo + SCFAs group respectively. DEGs labeled with the name were verified through western blot. (E) Gene set enrichment analysis (GSEA) in the BCCAo group versus BCCAo + FMT group and BCCAo group versus BCCAo + SCFAs group. Normalized enrichment score (NES) and nominal *p*‐value are indicated. BCCAo, bilateral common carotid artery occlusion; FMT, fecal microbiota transplantation; SCFAs, short‐chain fatty acids.

To identify the downstream signaling factors potentially involved in the effects of FMT and SCFAs on the hippocampus in response to CCH, the hippocampal transcriptome profile was evaluated in vivo. The results of Gene Ontology analysis identified five downregulated pathways related to histone modification, while some upregulated pathways, including neuronal cell body, regulation of cell growth, and synapse and energy metabolism, were associated with FMT and SCFAs (Figure 4A,C). Based on the Kyoto Encyclopedia of Genes and Genomes, the oxidative phosphorylation pathway was significantly correlated with FMT and SCFAs (Figure 4A,C). Furthermore, gene set enrichment analysis confirmed that oxidative phosphorylation, antioxidant activity, the synaptic vesicle cycle, mitochondrial respiratory chain complex I, ATPase activity, and oxidoreductase activity were markedly elevated after FMT and treatment with SCFAs (Figure 4E). Various DEGs were involved in these pathways, thus, some were further verified. Genes associated with the synapses (Gap43, Syp, and Stx1a), mitochondrial energy metabolism (Ndufb2 and Atp5mc1), and post‐ischemic inflammation and injury (Egr1)^32^, ^33^ were verified by western blot analysis and then included in a volcano plot (Figure 4B,D). These results indicate that inflammatory responses, neuronal cell growth, synapse, and energy metabolism might be prominent signaling pathways after FMT and treatment with SCFAs, thus warranting further investigations.

### 3.4 FMT and SCFAs mitigated CCH‐induced cognitive dysfunction

**FIGURE 5:**
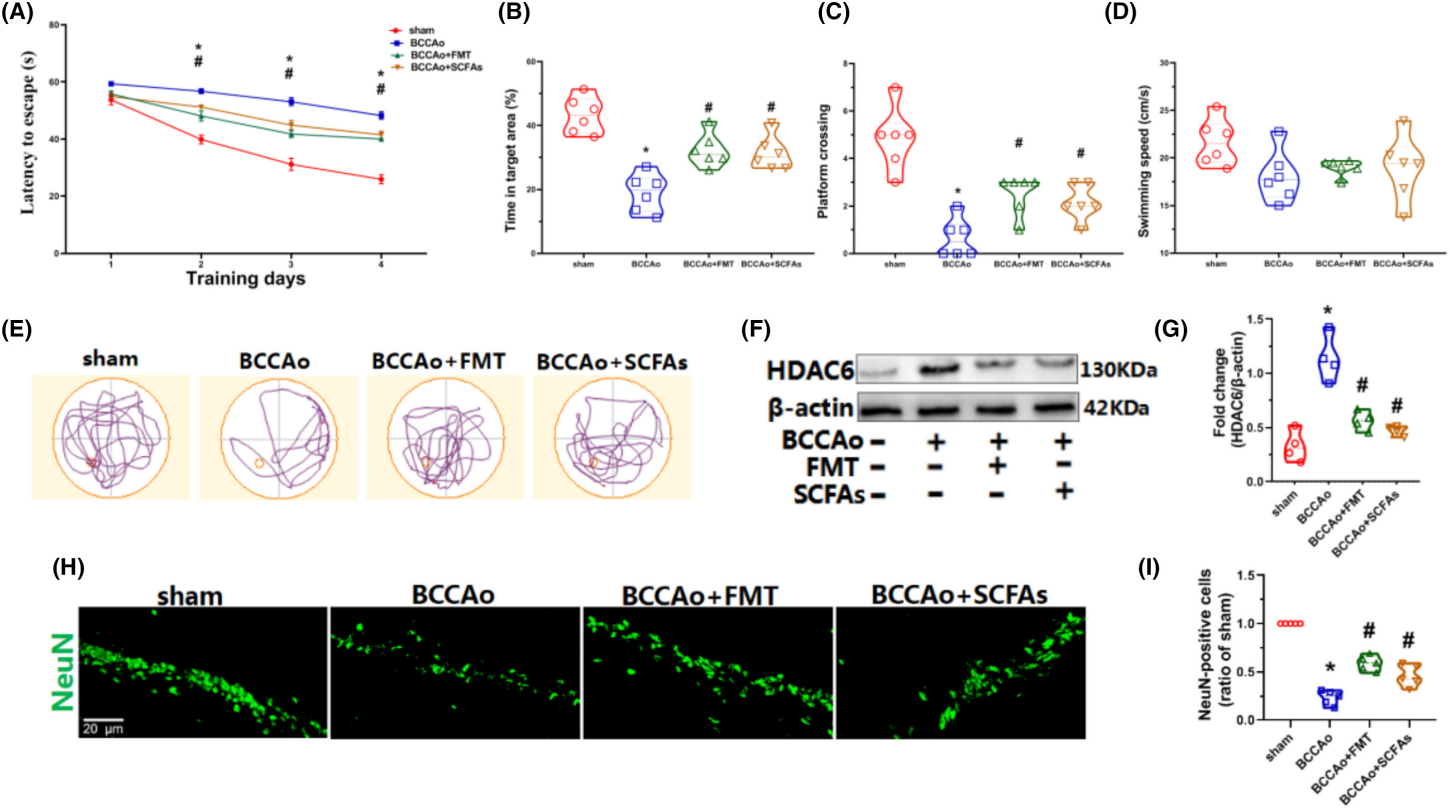
FMT and SCFAs treatment alleviated CCH‐induced cognitive dysfunction and neuronal loss by HDACs inhibition. (A) The average latency to escape to the hidden platform during the training days. (B) Time spent in the target quadrant among four groups during the probe trial. (C) Number of platform position crossings among the four groups during the probe trial. (D) The swimming speed among four groups during the probe trial. (E) Representative swimming paths among four groups during the probe trial. (F) Representative western blot for HDAC6 and β‐actin in hippocampus. (G) Relative optical density analysis for HDAC6 in hippocampus (*n* = 4 per group). (H) Representative immunofluorescence staining of NeuN in the hippocampal CA1 region (scale bars = 20 μm). (I) The relative level of NeuN‐positive cells in the hippocampal CA1 region (ratio of the sham group, *n* = 5 per group). The NeuN‐positive cells in the sham group are set to 1. Data are expressed as mean ± SEM. *p < 0.01 versus sham group. #*p* < 0.05 versus BCCAo group. FMT, fecal microbiota transplantation; SCFAs, short‐chain fatty acids.

As described in our previous study,^34^ CCH can lead to cognitive impairment. Based on the RNA‐seq results, some cognition‐related pathways, such as neuronal cell growth, synaptic plasticity, and inflammatory response, were responsive to FMT and SCFAs. Hence, the classic Morris water maze was used to observe cognitive function among the four groups. Rats in the BCCAo group showed more escape latency during the training period as well as less platform crossing and time in the target quadrant, thereby confirming impaired spatial learning ability. However, FMT and SCFAs strikingly rectified this phenomenon (Figure 5A–C). Furthermore, rats in the BCCAo group tended to aimlessly search for the platform, while those in the BCCAo + FMT and BCCAo + SCFAs groups displayed focal searching for the platform (Figure 5E). These results demonstrate the effects of FMT and SCFAs against cognitive impairment. A prior study showed that neuronal loss in the CA1 area of the hippocampus was strongly associated with cognitive impairment.^34^ Hence, immunocytochemical analysis of neuronal nuclear antigen expression was also conducted, which found that CCH‐induced neuronal loss was obviously prevented by FMT and SCFAs (Figure 5H,I), suggesting that SCFAs may influence microbiota‐gut‐brain interactions via free fatty acid receptors and inhibition of HDACs.^14^ However, free fatty acid receptors are rarely expressed in the hippocampus. Thus, protein levels of HDACs were further evaluated in the hippocampus. The results showed that CCH markedly upregulated Hdac6 protein levels, which were significantly downregulated by FMT and SCFAs (Figure 5F,G), indicating that this treatment strategy could ameliorate CCH‐induced cognitive dysfunction partly via the inhibition of HDACs.

### 3.5 FMT and SCFAs inhibited CCH‐induced synaptic impairment in the hippocampus

**FIGURE 6:**
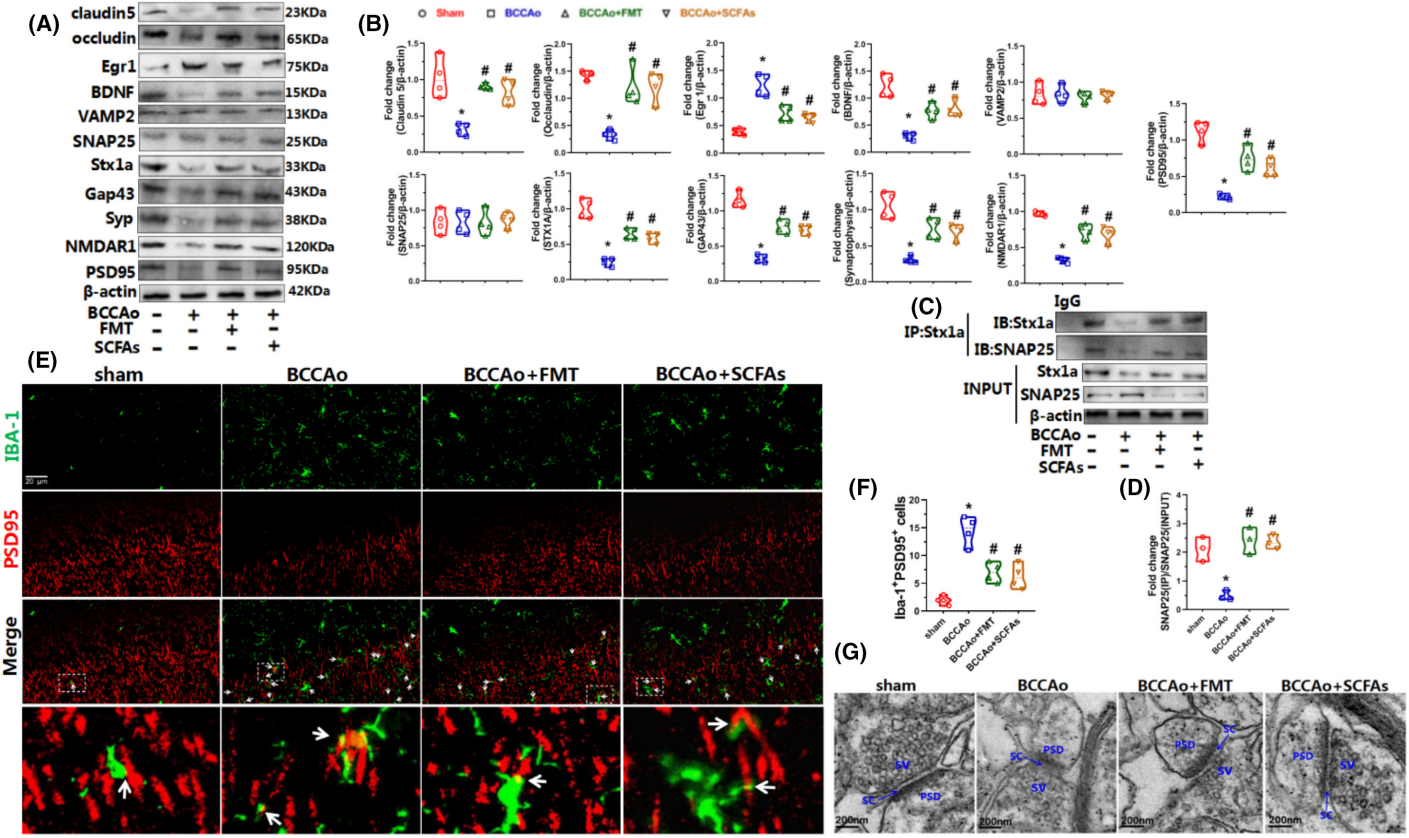
FMT and SCFAs treatment mitigated CCH‐induced hippocampal synaptic and BBB impairment. (A) Representative western blot of Egr1, BDNF, VAMP2, SNAP25, Stx1a, Gap43, Syp, NMDAR1, PSD95, occludin, claudin5, and β‐actin. (B) Relative optical density analysis of Egr1, BDNF, VAMP2, SNAP25, Stx1a, Gap43, Syp, NMDAR1, PSD95, occludin, and claudin5. (C) Representative immunoprecipitation (IP) of Stx1a, SNAP25, and β‐actin. (D) Relative abundance of SNAP25. The relative abundance of SNAP25 in the IP complex is determined by western blotting and compared to the level of SNAP25 in input. (E) Representative immunofluorescence staining of Iba‐1 and PSD95 in the hippocampal CA1 region (scale bars = 20 μm). Green puncta: Iba‐1; red puncta: PSD95; yellow puncta (indicated by white arrow): Iba‐1/PSD95 overlap. (F) The relative level of Iba‐1^+^PSD95^+^ cells. (G) The ultrastructure of synapses on the electron micrograph in the hippocampus CA1 region(scale bars = 200 nm). PSD: postsynaptic density; SC: synaptic cleft; SV: synaptic vesicle. Data are expressed as mean ± SEM (*n* = 4 per group). *p < 0.01 versus sham group. #*p* < 0.05 versus BCCAo group. FMT, fecal microbiota transplantation; SCFAs, short‐chain fatty acids.

Cerebral ischemia may lead to decreased plasticity of hippocampal synapses,^35^ which has been associated with cognitive dysfunction. The RNA‐seq results showed that synaptic pathways, such as synaptic vesicle, presynapse, postsynaptic density, neuron‐to‐neuron synapse, synapse organization, and synaptic vesicle transport, were upregulated after FMT and treatment with SCFAs. Hence, the ultrastructures of synapses in the CA1 region were compared among the four groups. The pre/postsynaptic membranes were unclear and blurred with wider synaptic clefts and lower postsynaptic density in the CCH group. However, FMT and SCFAs improved synaptic ultrastructure with clear pre/postsynaptic membranes and narrower synaptic clefts and greater postsynaptic density (Figure 6G). Then, the expression patterns of the presynaptic protein markers Syp and Gap43, postsynaptic protein markers PSD95 and NMDAR1, and synaptic vesicle docking/fusion‐related proteins Stx1a, SNAP25, and VAMP2 were assessed by western blot analysis. The results showed that the protein expression levels of Syp, Gap43, PSD95, NMDAR1, and Stx1a were significantly reduced in the BCCAo group, suggesting that CCH was associated with disruption to the pre/postsynaptic membranes as well as exocytosis of synaptic vesicles. Nevertheless, as compared to the BCCAo group, the protein levels of these markers were notably elevated in the BCCAo + FMT and BCCAo + SCFAs groups, indicating that synaptic transmission was restored, at least to a certain extent, by FMT and SCFAs (Figure 6A,B).

The soluble N‐ethylmaleimide‐sensitive factor attachment protein receptor (SNARE) complex, which consists of the vesicle‐SNARE protein VAMP2, located on synaptic vesicles, and target‐SNARE proteins, including Stx1a and SNAP25, located on the presynaptic membrane, promotes membrane fusion, leading to the release of synaptic neurotransmitters.^36^ Interestingly, CCH markedly decreased protein levels of Stx1a, but not VAMP2 or SNAP25 (Figure 6A,B). To determine whether the SNARE complex is affected by CCH, IP analysis was conducted with a Stx1a antibody to assess the binding of Stx1a with SNAP25. In addition, western blot analysis was performed to determine the protein level of SNAP25 in the Stx1a/SNAP25 complexes. The IP results showed that the ratio of SNAP25 in IP and SNAP25 in input was significantly downregulated by CCH, illustrating weakened binding of Stx1a and SNAP25 in response to CCH. However, this tendency was reversed by FMT and SCFAs, suggesting the prevention of CCH‐induced dissociation of SNAP25 from Stx1a (Figure 6C,D).

### 3.6 FMT and SCFAs reshaped microglial polarization patterns and relieved synaptic engulfment in hippocampal tissues after CCH

**FIGURE 7:**
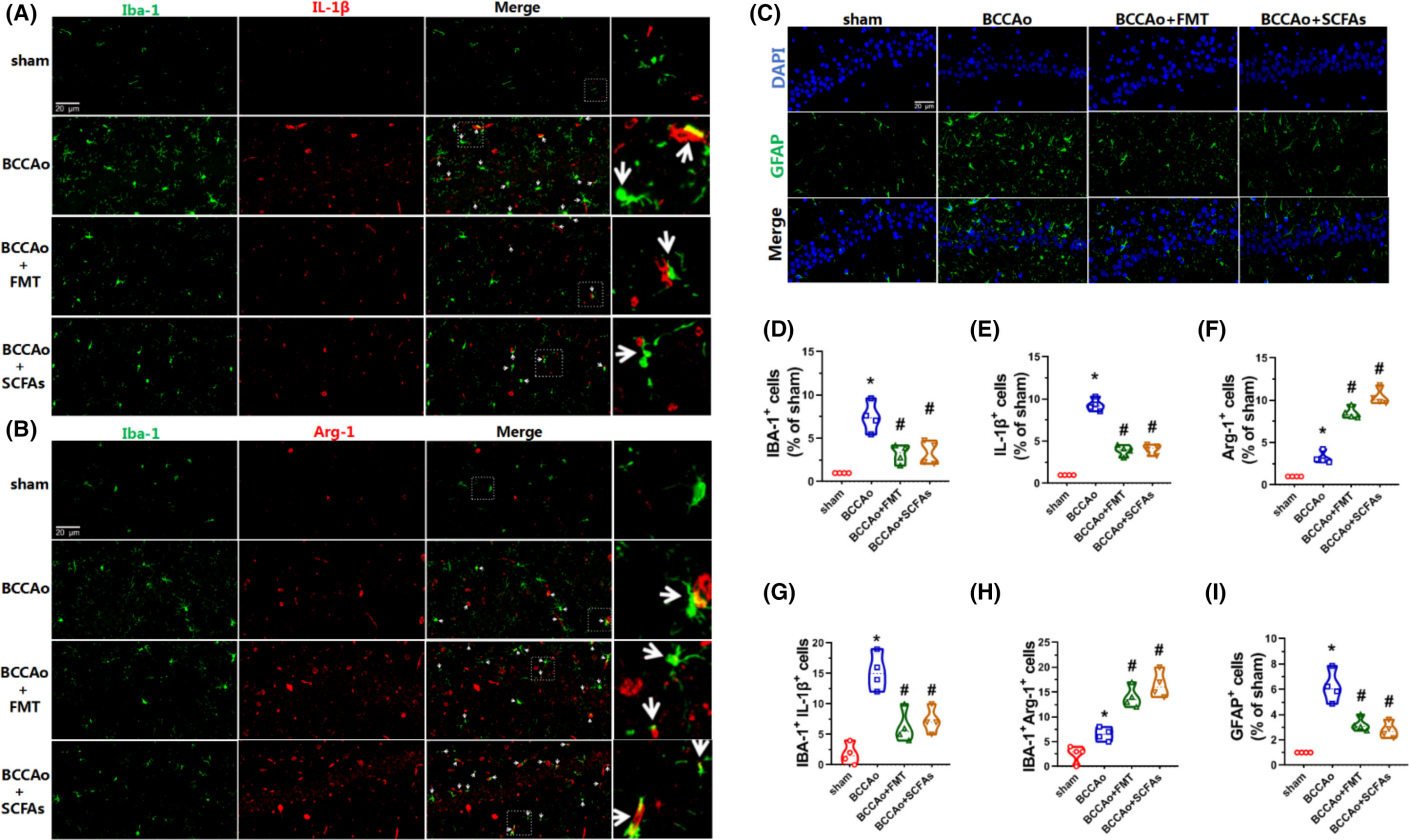
FMT and SCFAs treatment relieve CCH‐induced hippocampal microglia and astrocyte activations. (A) Representative immunofluorescence staining of Iba‐1 and IL‐1β in the hippocampal CA1 region (scale bars = 20 μm). Green puncta: Iba‐1; red puncta: IL‐1β; yellow puncta (indicated by white arrow): Iba‐1/IL‐1β overlap. (B) Representative immunofluorescence staining of Iba‐1 and Arg‐1 in the hippocampal CA1 region (scale bars = 20 μm). Green puncta: Iba‐1; red puncta: Arg‐1; yellow puncta (indicated by white arrow): Iba‐1/Arg‐1 overlap. (C) Representative immunofluorescence staining of GFAP in the hippocampal CA1 region (scale bars = 20 μm). Green puncta: GFAP; blue puncta: DAPI. (D) The relative level of Iba‐1^+^ cells (ratio of the sham group). The Iba‐1‐positive cells in the sham group are set to 1. (E) The relative level of IL‐1β^+^ cells (ratio of the sham group). The IL‐1β‐positive cells in the sham group are set to 1. (F) Relative level of Arg‐1^+^ cells (ratio of the sham group). The Arg‐1‐positive cells in the sham group are set to 1. (G) The relative level of Iba‐1^+^IL‐1β^+^ cells. (H) Relative level of Iba‐1^+^Arg‐1^+^ cells. (I) The relative level of GFAP^+^ cells (ratio of the sham group). The GFAP‐positive cells in the sham group are set to 1. Data are expressed as mean ± SEM (*n* = 4 per group). *p < 0.01 versus sham group. #*p* < 0.05 versus BCCAo group. BCCAo, bilateral common carotid artery occlusion; FMT, fecal microbiota transplantation; SCFAs, short‐chain fatty acids.

Egr1 is a transcription factor that regulates the differentiation and activation of macrophages, which is closely related to the activation of microglia and astrocytes.^37^, ^38^ In the present study, mRNA expression of Egr1 was sharply upregulated by CCH and reversed by FMT and treatment with SCFAs. Therefore, western blot analysis was conducted to measure the protein levels of Egr1, which confirmed the mRNA results (Figure 6A,B). Microglial activation and polarization were subsequently evaluated by immunofluorescence analysis of Iba‐1, IL‐1β, and Arg‐1 as markers of microglia and the M1 and M2 subtypes, respectively. The proportion of IL‐1β‐positive cells was upregulated in the CCH group, but not the FMT and SCFAs groups. Nevertheless, the proportion of Arg‐1‐positive cells was downregulated after FMT and treatment with SCFAs (Figure 7A,B,E,F). The ratios of Iba‐1^+^IL‐1β^+^ and Iba‐1^+^Arg‐1^+^cells were markedly increased by CCH, suggesting that CCH induced microglial activation and differentiation to the M1 subtype. After FMT and treatment with SCFAs, the proportions of Iba‐1^+^ and Iba‐1^+^IL‐1β^+^ cells had significantly decreased, while the proportion of Iba‐1^+^Arg‐1^+^cells had significantly increased, confirming that FMT and treatment with SCFAs inhibited microglial activation and accelerated differentiation to the M2 subtype (Figure 7A,B,D,G,H). In addition, astrocytic activation was assessed by immunofluorescence of glial fibrillary acidic protein as a marker of astrocytes, which demonstrated the same tendency as microglial activation (Figure 7C,I). In brief, FMT and treatment with SCFAs inhibited expression of the transcription factor Egr1 and activation of microglia and astrocytes, while promoting differentiation from the M1 to the M2 phenotype, demonstrating anti‐neuroinflammatory effects. Furthermore, M1 pro‐inflammatory microglia contribute to the impairment of BBB integrity via the regulation of tight junction proteins.^39^, ^40^ Thus, the protein expression levels of tight junction proteins, including claudin 5 and occluding, were measured as markers of BBB integrity. The results showed that the expression levels of claudin 5 and occludin were notably reduced by CCH, while these trends were reversed by FMT and treatment with SCFAs, suggesting that the treatment strategy maintained BBB integrity (Figure 6A,B). In addition, to confirm whether there is a link between microgliosis and synapse loss in response to CCH, the expression patterns of Iba‐1 and PSD95 in hippocampal tissues were investigated. Other than the increase in Iba‐1‐positive puncta and decrease in PSD95‐positive puncta, CCH increased PSD95‐positive puncta enveloped by Iba‐1‐positive puncta, suggesting that CCH induced engulfment of the hippocampal synapses. However, colocalization of Iba‐1 and PSD95 had decreased after FMT and treatment with SCFAs (Figure 6E,F), indicating protective effects against microglia‐mediated engulfment of synapses.

### 3.7 FMT and SCFAs prevented injury to the hippocampal mitochondria

**FIGURE 8:**
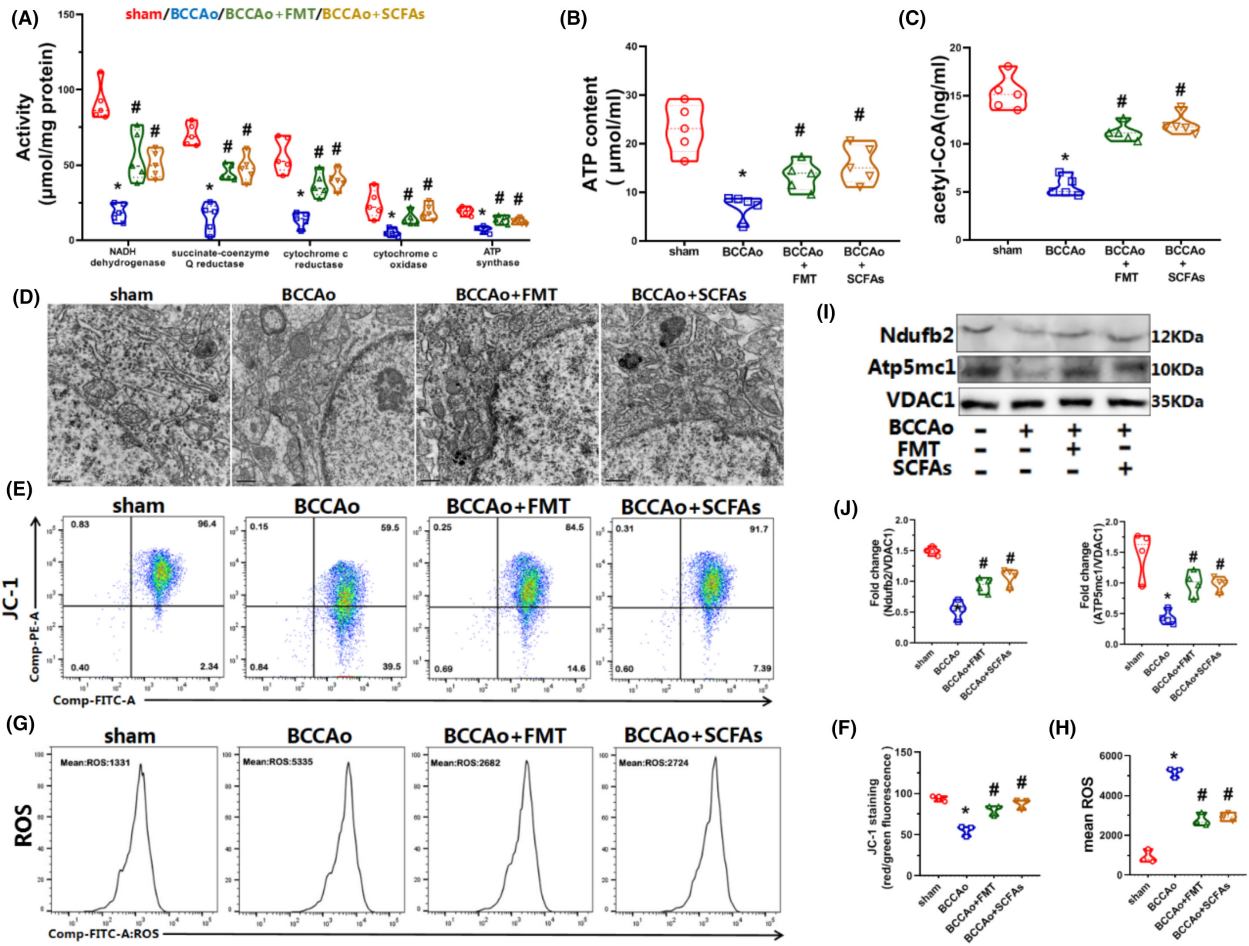
FMT and SCFAs treatment relieve CCH‐induced hippocampal mitochondrial dysfunction. (A) The activities of mitochondrial electron transport chain Complex I‐V (*n* = 5 per group). (B) The content of ATP (*n* = 5 per group). (C) The content of acetyl‐CoA (*n* = 5 per group). (D) Representative electron micrographs of mitochondria in the hippocampus CA1 region (scale bars = 0.5 μm). (E) Representative plot of JC‐1 staining for mitochondrial membrane potential analyzed by flow cytometry. (F) Bar charts of the percentage of mitochondria with normal membrane potential according to JC‐1 staining (*n* = 3 per group). (G) Representative plot of DHE staining for ROS analyzed by flow cytometry. (H) Bar charts of mean ROS according to DHE staining (*n* = 3 per group). (I) Representative western blot of Ndufb2, Atp5mc1, and VDAC1. (J) Relative optical density analysis of Ndufb2 and Atp5mc1 (*n* = 4 per group). Data are expressed as mean ± SEM. *p < 0.05 versus sham group. #*p* < 0.05 versus BCCAo group. BCCAo, bilateral common carotid artery occlusion; FMT, fecal microbiota transplantation; SCFAs, short‐chain fatty acids.

Based on the RNA‐seq results, upregulated pathways in response to CCH after FMT and treatment with SCFAs were associated with the ATP metabolic process, mitochondrial respiratory chain, and electron transfer activity (Figure 4A,C). Therefore, the mitochondrial ultrastructure was assessed by transmission electron microscopy, which revealed abnormal mitochondrial swelling, vague cristae, and membrane fragmentation in the CCH group, which were substantially reduced in the FMT and SCFAs groups (Figure 8D). Furthermore, mitochondrial membrane potential was assessed by flow cytometry of JC‐1 staining. The results demonstrated that the number of mitochondria with normal membrane potential was markedly decreased in the CCH group as compared to the sham group but notably increased after FMT and treatment with SCFAs (Figure 8E,F). Moreover, to determine whether disrupted hippocampal mitochondrial energy metabolism was associated with CCH and the potential effects of FMT and treatment with SCFAs, mitochondrial ETC complex I–V activities and ATP content in hippocampal tissues were measured. The results showed that CCH decreased the activities of mitochondrial ETC complexes I–V, which eventually led to lower ATP generation, confirming that CCH interfered with mitochondrial oxidative phosphorylation. However, these phenomena were reversed by FMT and treatment with SCFAs (Figure 8A,B). Inefficient oxidative phosphorylation is reported to promote the accumulation of ROS, resulting in mitochondrial dysfunction.^41^ Subsequently, mitochondrial ROS production was assessed by flow cytometry of DHE staining. The amount of ROS was notably elevated by CCH but strikingly reduced by FMT and treatment with SCFAs (Figure 8G,H), thereby demonstrating protective effects against CCH‐induced dysregulation of mitochondrial energy metabolism.

To further explore the potential mechanisms underlying the effects of FMT and SCFAs against CCH‐induced mitochondrial dysfunction, the protein expression levels of two significant DEGs, Ndufb2 and Atp5mc1, subunits of ETC complexes I and V, were measured. The results showed that CCH dramatically downregulated the protein levels of Ndufb2 and Atp5mc1, corroborating that mitochondrial ETC complexes I and V play important roles in CCH‐induced dysfunction of mitochondrial oxidative phosphorylation. Nevertheless, decreased protein expression of Ndufb2 and Atp5mc1 was alleviated by FMT and SCFAs, indicating protective effects against chronic CI via reprogramming mitochondrial metabolism (Figure 8I,J). In addition, acetate, which was significantly increased after FMT and treatment with SCFAs, is a major nutrient involved in acetyl‐CoA metabolism.^42^ Acetyl‐CoA, which is reported to increase the tricarboxylic acid cycle, oxidative phosphorylation, and ATP production, plays central roles in energy production.^43^ Therefore, the acetyl‐CoA content in the hippocampus was evaluated with an ELISA. As expected, CCH markedly downregulated the hippocampal content of acetyl‐CoA, which was reversed by FMT and treatment with SCFAs, suggesting that increased acetate production could improve the hippocampal content of acetyl‐CoA (Figure 8C).

## 4 DISCUSSION

The present study is the first to provide evidence that chronic CI can change the structure and composition of the GM, decrease fecal acetic and propionic acid contents, and reduce the hippocampal content of acetic acid. FMT and SCFAs relieved CCH‐induced GM dysbiosis by enriching the proportions of some bacterial groups of the GM, including *Akkermansia*, *Ruminococcus*, and *Turicibacter*, and substantially strengthen fecal acetic and propionic acid production and hippocampal acetic acid generation. Of note, *Ruminococcus_sp_N15_MGS_57* was the only intestinal bacteria closely related to acetic acid generation by comparison of the BCCAo vs. BCCAo + FMT groups and BCCAo vs. BCCAo + SCFAs groups, confirming that *Ruminococcus_sp_N15_MGS_57* and acetic acid might be responsible for the effects of FMT and SCFAs in response to CCH. FMT and SCFAs protected against CCH‐induced neuroinflammation by upregulation of BBB‐related tight junction proteins, inhibition of microglial and astrocytic activation, and switching microglial polarization from the M1 to the M2 subtype. Furthermore, FMT and SCFAs protected against CCH‐induced cognitive dysfunction via inhibition of microglia‐mediated synaptic loss, prevention of neuronal loss, and maintaining normal function of the pre/postsynaptic membranes and fusion of synaptic vesicles. In addition, FMT and SCFAs restored hippocampal mitochondrial function by normalizing mitochondrial membrane potential, decreasing ROS accumulation, and enhancing mitochondrial ETC and oxidative phosphorylation. FMT and SCFAs may exert neuroprotective effects via HDACs inhibition. Collectively, these findings support hippocampal neuroprotection by FMT and SCFAs against chronic CI.

The concept of the influence of the GM on brain function is still in the primary stage. A study of patients with cirrhosis illustrated that *Ruminococcus* were associated with good cognitive function independent of clinical variables,^44^ whereas another study of patients with Parkinson's disease demonstrated that an abundance of *Ruminococcus* was negatively correlated with cognitive function.^45^ Furthermore, two randomized controlled trials of older adults reported conflicting results on the effects of *Ruminococcus* on cognitive function. One found that *Ruminococcus* were correlated with cognitive function, while the other repudiated this association.^46^, ^47^ The above results suggest that the influence of *Ruminococcus* on cognitive function is very complex and dependent on disease status. Accumulated evidence has revealed that decreased acetic acid production is associated with microglial activation and cognitive decline.^48^, ^49^, ^50^ Suppression of the ERK/JNK/NF‐κB pathway, prebiotic changes in fecal SCFAs, and vagus nerve stimulation might be the mechanisms underlying the above effects. Consistent with previous studies, CCH induced significant reductions in the acetic acid contents of feces and hippocampal tissues, leading to hippocampal microglial activation and polarization as well as cognitive impairment, which were reversed by FMT and SCFAs. Moreover, *Ruminococcus* mainly metabolize inositol and sugar alcohols and utilize formate to produce acetic acid.^51^ In the present study, Spearman correlation analysis further confirmed that *Ruminococcus_sp_N15_MGS_57* was positively correlated with the acetic acid generation, indicating that the abundance of *Ruminococcus_sp_N15_MGS_57* might influence the hippocampal neuroprotective effects of FMT and SCFAs in response to chronic CI. Previous studies have shown that *Akkermansia* and *Turicibacter* could ameliorate neuronal injury.^52^, ^53^, ^54^ Although there was no significant relationship with SCFAs, *Akkermansia* and *Turicibacter* may affect brain function via other GM‐related metabolic factors, such as isoflavones, phytoestrogens, and phosphotransferases,^54^, ^55^ thus, warranting further investigations.

Impaired synaptic plasticity and abnormal synaptic vesicle release have been implicated in the neuropathology of CI‐induced cognitive decline.^56^, ^57^ Acetic acid supplementation was shown to improve synaptic plasticity in the hippocampus via the inhibition of HDACs in a mouse model of depression.^24^ In response to CI, butyric acid ameliorated acute CI‐induced impairment of synaptic plasticity.^58^, ^59^ In line with the results of previous studies, FMT and SCFAs strikingly upregulated the CCH‐induced reduced expression of pre/postsynaptic proteins, including Syp, Gap43, PSD95, and NMDAR1, confirming the protective effects of FMT and SCFAs by maintaining synaptic homeostasis, which was also verified by observations of the synapse ultrastructure. Furthermore, synaptic vesicles secrete neurotransmitters by fusion with the presynaptic membrane, which is the central step in neuronal communication.^60^ Previous studies found that impaired synaptic vesicle fusion in the presynaptic area contributed to cognitive dysfunction after ischemia‐reperfusion injury.^24^ Accordingly, in the present study, synaptic vesicle fusion in the formation of SNARE complexes was disrupted in a state of chronic CI. Stx1a, a target‐SNARE protein, is the requisite component of the SNARE complex for synaptic vesicular release. Synaptic transmission cannot proceed without Stx1a. In the present study, FMT and SCFAs reversed CCH‐induced downregulation of Stx1a protein levels. To the best of our knowledge, the present study provides the first evidence that Stx1a is a key regulator of chronic CI and the effectiveness of FMT and SCFAs. Interestingly, there was no significant change in the expression levels of the two SNARE proteins VAMP2 and SNAP25. However, CCH promoted dissociation of SNAP25 from the Stx1a/SNAP25 complex, which obstructed target‐SNARE formation, while FMT and SCFAs inhibited dissociation of SNAP25 from Stx1a, indicating that FMT and SCFAs strengthened synaptic transmission by maintaining the SNARE complexes. Stx1a and the target‐SNARE protein complex are potential regulatory targets in microbiota‐based treatment for chronic CI. Taken together, these findings suggest that synaptic plasticity and synaptic vesicle fusion and release could be mechanisms employed by FMT and SCFAs against chronic CI.

Microglia are the innate immune effector cells in the CNS and maintain brain homeostasis. Microglial activation is a key pathological component of chronic CI,^61^ which is associated with neuronal loss.^62^ SCFAs were shown to convey anti‐hippocampal neuroinflammatory effects in mice fed a high‐fructose diet.^63^ In the present study, CCH impaired the function of BBB‐related tight junction proteins, inhibited activation of the microglia and astrocytes, increased the ratio of M1/M2 microglia, and promoted neuronal loss in the hippocampus, confirming hippocampal neuroinflammation in a state of chronic CI. However, FMT and SCFAs protected BBB integrity by upregulating the levels of tight junction proteins, including occludin and claudin 5, and suppressed neuroinflammation by inhibiting activation of microglia and astrocytes and promoting the switch from the pro‐inflammatory M1 subtype to the anti‐inflammatory M2 subtype, which subsequently reversed neuronal loss. Furthermore, microglial engulfment of presynaptic terminals resulted in synaptic loss and cognitive dysfunction.^64^ Intriguingly, in a state of chronic CI, CCH‐induced activated microglia had co‐localized with the postsynaptic marker PSD95, indicating engulfment of postsynaptic, rather than presynaptic, terminals in the hippocampus, which was significantly mitigated by FMT and SCFAs. This finding is in accordance with another study of the association of the GM and metabolites of SCFAs,^65^ suggesting that engulfment of postsynaptic terminals might be one of the important mechanisms of the GM and SCFAs against synaptic loss.

Knockout or inhibition of Egr1, a zinc finger transcription factor, suppressed activation of microglia and astroglia, and protected dopaminergic neurons in a mouse model of Parkinson's disease, confirming the crucial role of astrocyte‐induced increased Egr1 expression in both neuronal death and neuroinflammatory responses.^38^ On the other hand, Egr1 induction in neurons is tightly associated with many forms of neuronal activity, demonstrating the protective effects for synaptic and neuronal plasticity.^66^ Egr1 functions in the CNS are complex and regulated by a wide variety of environmental factors.^67^ In a state of chronic CI, astroglial activation was enhanced by CCH and reversed by FMT and SCFAs. The protein expression profile of Egr1 was the same as that of astrogliosis, suggesting that Egr1 is mainly induced by astrocytes in a state of chronic CI, illustrating pro‐neuroinflammation activities.

The primary physiological function of the mitochondria is the generation of ATP via mitochondrial ETC‐mediated oxidative phosphorylation.^68^ Inefficient oxidative phosphorylation leads to the accumulation of ROS and subsequent mitochondrial dysfunction.^42^ In the present study, impaired mitochondrial ETC and oxidative phosphorylation resulted in reduced ATP production, excessive ROS generation, and mitochondrial dysfunction in a state of chronic CI. Acetic acid is a major nutrient that supports the metabolism of acetyl‐CoA,^42^ which plays important roles in oxidative phosphorylation and mitochondrial ATP production in a state of chronic CI.^69^ FMT and SCFAs increased the contents of acetic acid, acetyl‐CoA, and ATP, as well as the activities of mitochondrial ETC complexes I–V, thereby alleviating mitochondrial dysfunction. These results indicate that increased hippocampal acetic acid and acetyl‐CoA may, at least in part, contribute to the beneficial effects of FMT and SCFAs against chronic CI‐induced dysfunction of mitochondrial energy metabolism. Further investigations found that the protein levels of Ndufb2 and Atp5mc1 were significantly altered by FMT and SCFAs, suggesting that mitochondrial ETC complexes I and V might be the major sites of oxidative phosphorylation regulated by FMT and SCFAs. Furthermore, given the lack of a suitable cell model for chronic CI, future studies are needed to investigate mitochondrial oxygen consumption in different CNS cells, including neurons, microglia, and astrocytes, in a state of chronic CI.

In conclusion, previous studies confirmed the potential influence of the microbiota‐gut‐brain axis on the treatment outcomes of ischemic stroke and neurodegenerative disorders.^70^, ^71^ This study is the first to provide evidence that chronic CI‐induced cognitive dysfunction involves alterations to the gut‐hippocampus axis, GM dysbiosis, and reductions in SCFAs. FMT and SCFAs exerted neuroprotective effects by inhibiting microglia‐mediated neuroinflammation and synaptic loss while maintaining normal fusion and release of synaptic vesicles, improving hippocampal synaptic plasticity, and relieving impaired mitochondrial ETC‐mediated oxidative phosphorylation by increasing the intestinal content of *Ruminococcus_sp_N15_MGS_57* and the metabolite acetic acid. These findings highlight a GM‐based strategy to increase the contents of SCFAs to reduce neurological injury associated with chronic CI.

## AUTHOR CONTRIBUTIONS

Shao‐Hua Su, Ming Chen, and Jian Hai designed the research. Ming Chen, Yi‐Fang Wu, and Jun Sun performed the experiments. Qi Lin and Da‐Peng Wang analyzed data. CM, Yi‐Fang Wu, Qi Lin, Da‐Peng Wang, and Jun Sun participated in figure preparation. Shao‐Hua Su and Jian Hai drafted the manuscript. All authors read the article and approved the manuscript.

## CONFLICT OF INTEREST

The authors declare that they have no conflicts of interest concerning this article.

## Data Availability

The 16S rRNA‐seq and RNA‐seq raw data that support the findings of this study have been deposited into NCBI BioProject with accession numbers PRJNA869931 and PRJNA827266. The other data are available from the corresponding author upon reasonable request.
